# *O*-GlcNAc-Specific Antibody CTD110.6 Cross-Reacts with *N*-GlcNAc_2_-Modified Proteins Induced under Glucose Deprivation

**DOI:** 10.1371/journal.pone.0018959

**Published:** 2011-04-19

**Authors:** Takahiro Isono

**Affiliations:** Central Research Laboratory, Shiga University of Medical Science, Otsu, Shiga, Japan; New York State Institute for Basic Research, United States of America

## Abstract

Modification of serine and threonine residues in proteins by *O*-linked β-*N*-acetylgulcosamine (*O*-GlcNAc) glycosylation is a feature of many cellular responses to the nutritional state and to stress. *O*-GlcNAc modification is reversibly regulated by *O*-linked β-*N*-acetylgulcosamine transferase (OGT) and β-D-*N*-acetylgulcosaminase (*O*-GlcNAcase). *O*-GlcNAc modification of proteins is dependent on the concentration of uridine 5′-diphospho-*N*-acetylgulcosamine (UDP-GlcNAc), which is a substrate of OGT and is synthesized via the hexosamine biosynthetic pathway. Immunoblot analysis using the *O*-GlcNAc-specific antibody CTD110.6 has indicated that glucose deprivation increases protein *O*-GlcNAcylation in some cancer cells. The mechanism of this paradoxical phenomenon has remained unclear. Here we show that the increased glycosylation induced by glucose deprivation and detected by CTD110.6 antibodies is actually modification by *N*-GlcNAc_2_, rather than by *O*-GlcNAc. We found that this induced glycosylation was not regulated by OGT and *O*-GlcNAcase, unlike typical *O*-GlcNAcylation, and it was inhibited by treatment with tunicamycin, an *N*-glycosylation inhibitor. Proteomics analysis showed that proteins modified by this induced glycosylation were *N*-GlcNAc_2_-modified glycoproteins. Furthermore, CTD110.6 antibodies reacted with *N*-GlcNAc_2_-modified glycoproteins produced by a yeast strain with a ts-mutant of ALG1 that could not add a mannose residue to dolichol-PP-GlcNAc_2_. Our results demonstrated that *N*-GlcNAc_2_-modified glycoproteins were induced under glucose deprivation and that they cross-reacted with the *O*-GlcNAc-specific antibody CTD110.6. We therefore propose that the glycosylation status of proteins previously classified as *O*-GlcNAc-modified proteins according to their reactivity with CTD110.6 antibodies must be re-examined. We also suggest that the repression of mature N-linked glycoproteins due to increased levels of *N*-GlcNAc_2_-modifed proteins is a newly recognized pathway for effective use of sugar under stress and deprivation conditions. Further research is needed to clarify the physiological and pathological roles of *N*-GlcNAc_2_-modifed proteins.

## Introduction

**Figure 1:**
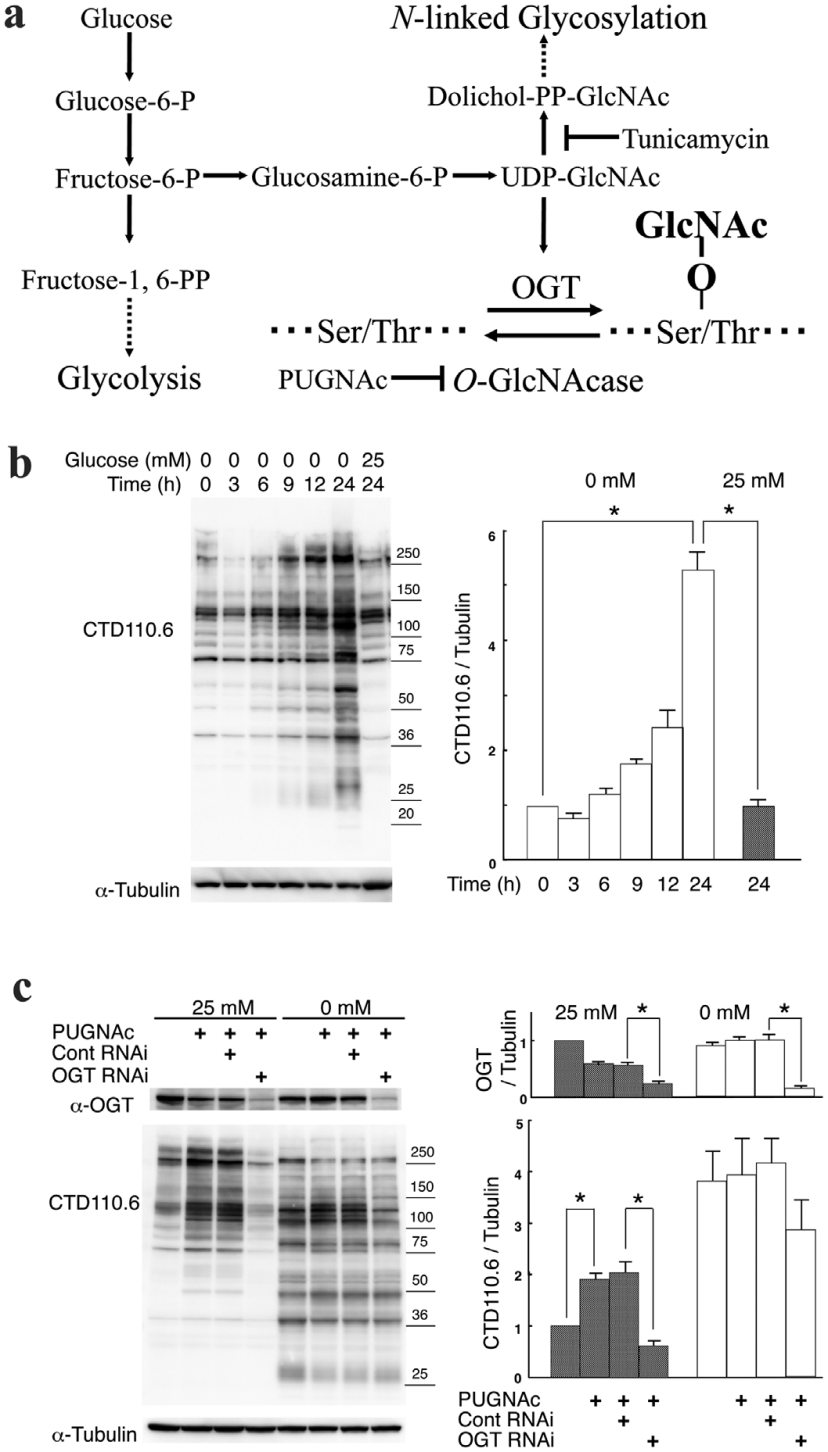
The glycosylation induced under glucose deprivation was not regulated by OGT and *O*-GlcNAcase. **a**, The hexosamine biosynthetic pathway and *O*-GlcNAcylation. **b**, An immunoblot showing CTD110.6 antibody reaction over the time course during incubation of T24 cells in glucose deprivation medium (0 mM glucose). An anti-α-tubulin antibody was used as an internal control. The right panel shows a quantitative analysis of reactivity with the CTD110.6 antibody, normalized to the anti-α-tubulin antibody signal of the time 0 samples. Error bars represent standard error from three experiments. * represents p<0.001. **c**, The effects of treatment with siRNA against *OGT* and with PUGNAc on expression levels of proteins that reacted with CTD110.6 antibodies under glucose deprivation. The left panel shows an immunoblot for CTD110.6, anti-OGT, and anti-α-tubulin antibodies. The right panel shows a quantitative analysis of reactivity with the CTD110.6 antibody and with an anti-OGT antibody, normalized to the anti-α-tubulin signal for untreated samples in high-glucose medium. Error bars represent standard error from three experiments. * represents p<0.05.

We previously screened proteins as tumor markers for bladder cancer by proteomic analysis of cancerous and health tissues and identified some proteins, calreticulin et al, as markers [1]–[3]. We next attempted to screen for proteins that were key molecules in metastasis and invasiveness by proteomic analysis of bladder carcinoma cell line T24 cells. The T24 cells were modified by introduction of a tumor suppressor gene, after which they lost their invasiveness activity [4]. However, we could not find protein expression patterns that differed between these cell types. Therefore, we devised two improvements: application of focused proteomics and use of cells under stress conditions. Application of focused proteomics involves screening of minor key proteins. The usual culture medium has very rich nutrients and does not reflect to the in vivo environment. We examined changes in the *O*-linked β-*N*-acetylgulcosamine (*O*-GlcNAc)-glycosylated proteins in T24 cells under glucose deprivation. *O*-GlcNAc-modification of proteins is a feature of many cellular responses to the nutritional state and to stress [5]–[7]. *O*-GlcNAc is attached to the protein backbone by enzymatic addition of the *N*-acetylgulcosamine (GlcNAc) moiety of uridine 5′-diphospho (UDP)-GlcNAc to the hydroxyl oxygen of serines or threonines by the *O*-linked β-*N*-acetylgulcosamine transferase (OGT). *O*-GlcNAc glycosylated proteins can be reversiblely deglycosylated by β-D-*N*-acetylgulcosaminase (*O*-GlcNAcase). These reversible *O*-GlcNAc glycosylations are distinct from stable, complex *N*-linked glycosylations of membrane or secreted proteins, which takes place in the lumen of the endoplasmic reticulum and in the Golgi apparatus. *O*-GlcNAc modification of proteins is dependent on the concentration of UDP-GlcNAc, which is synthesized by the hexosamine biosynthetic pathway (HBP, Figure 1a) [8]. Glucose deprivation induced increased expression of proteins that reacted with the *O*-GlcNAc specific antibody CTD110.6, despite the decreased concentration of UDP-GlcNAc. These paradoxical phenomena were previously reported for the human hepatocellular carcinoma cell line HepG2 cells, mouse neuroblastoma cell line Neuro-2a cells, and some cancerous cells [9]–[12]. In this study, we attempted to identify and characterize the proteins in T24 cells that reacted with the *O*-GlcNAc-specific antibody CTD110.6 under glucose deprivation. Our results demonstrated that *N*-GlcNAc_2_-modified glycoproteins were induced under glucose deprivation and that they cross-reacted with the *O*-GlcNAc-specific antibody CTD110.6.

## Results

When T24 human bladder cancer cells were subjected to glucose deprivation, the expression of proteins detected by the *O*-GlcNAc specific antibody CTD110.6 increased more than 5-fold (Figure 1b), as previously reported [9]–[12]. The expression of basal proteins that reacted with CTD110.6 antibodies in high-glucose medium (25 mM glucose) decreased 3 h after glucose deprivation initiated by incubation of the cells in medium with 0 mM glucose, but the signals returned to the original basal levels 6 h after glucose deprivation was initiated. Expression of the newly induced proteins, which reacted with CTD110.6 antibodies only under glucose deprivation conditions, detected at the 6 h time point, continued to increase until 24 h after the treatment, become major part of proteins that reacted with CTD110.6 antibodies 24 h after the treatment (Figure 1b). These results suggested that two kinds of proteins, the basal proteins and the induced proteins, were produced under glucose deprivation conditions.

We next examined the effects of treating the cells with siRNA specific for *OGT* and with PUGNAc, an inhibitor of *O*-GlcNAcase, on expression of proteins that reacted with CTD110.6 antibodies under glucose deprivation (Figure 1c). In the high-glucose medium, expression of the basal proteins increased following treatment with PUGNAc, and the signal clearly decreased following knockdown of *OGT* with siRNA, as expected. When the cells were incubated in glucose deprivation medium, the responses of basal protein expression levels to treatment with PUGNAc and knockdown with siRNA were similar in the high-glucose medium. However, the induced protein expression levels did not increase following treatment with PUGNAc and their levels did not decrease following siRNA knockdown, unlike the basal proteins. The induced proteins also prevented the reactivity with CTD110.6 antibodies upon addition of 10 mM GlcNAc, like the basal proteins (Figure S1), but they did not react with another *O*-GlcNAc specific antibody, RL-2, unlike the basal proteins (Figure S2). Then the induced proteins did not react clearly with the Click-It *O*-GlcNAc detection system, though the basal proteins reacted strongly with this system (Figure S3). These results suggested that the induced proteins differed from the typical *O*-GlcNAc-modified proteins, which included the basal proteins.

**Figure 2:**
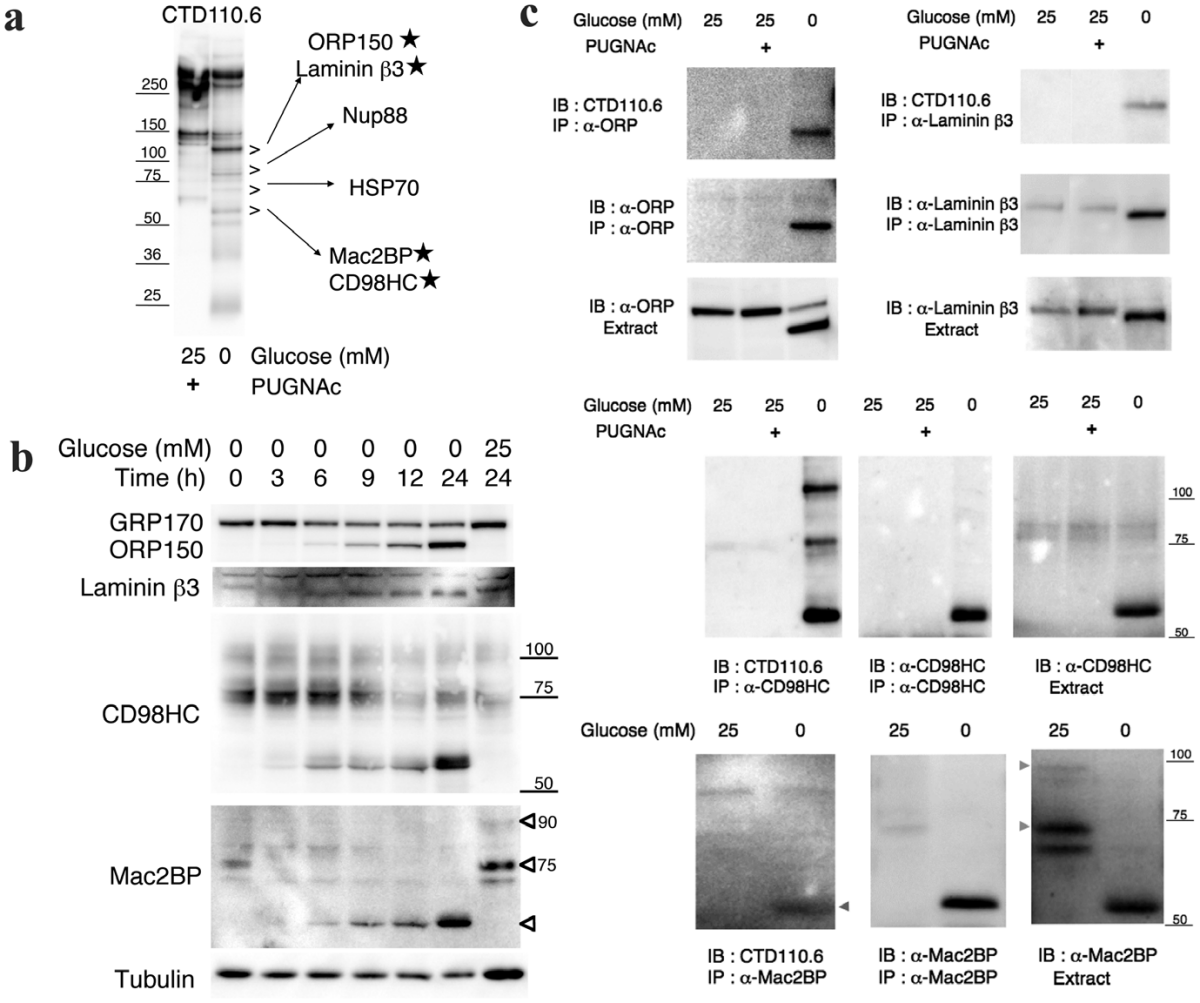
The identification and validation of proteins induced under glucose deprivation that reacted with CTD110.6 antibodies. **a**, The induced proteins were detected by CTD110.6 antibody. The > marks indicate the area excised for LC/MS/MS. The identified proteins are indicated. **b**, An immunoblot showing the four proteins induced by glucose deprivation. The incubation times for in high-glucose medium (25 mM glucose) or in glucose deprivation medium (0 mM glucose) are indicated. An anti-α-tubulin antibody was used as an internal control. **c**, Immunoblots showing immunoprecipitation samples of the four induced proteins.

The induced proteins were next analyzed by LC/MS/MS. Six proteins were identified, as shown in Figure 2a and Table S1. Two of the six proteins, Nup88 and HSP70, are common *O*-GlcNAc-modified proteins [5], and they may constitute some of the basal proteins. The other four proteins (ORP150, Laminin β3, Mac2BP, and CD98HC) are glycoproteins with *N*-linked sugars, and they had not been identified previously as *O*-GlcNAc-modified proteins.

We carried out the validations of the four proteins identified by their reactivity with CTD110.6 antibodies following glucose deprivation of T24 cells by immunoblot (Figure 2b) and immunoprecipitation analysis (Figure 2c). Immunoblot analysis using anti-ORP150 antibodies showed a decrease in the glycosylated form, GRP170, in the high-glucose medium, while levels of the lower molecular weight form corresponding to non-glycosylated ORP150 increased under glucose deprivation. Immunoblot analysis with anti-Laminin β3 antibodies showed that Laminin β3 also shifted to the lower molecular weight form in glucose deprivation medium. The bands seen for the lower molecular weight forms of ORP150 and Laminin β3 corresponded to the 120 kDa bands previously identified as proteins based on their reaction with CTD110.6 antibodies under glucose deprivation. Immunoblot analysis with anti-CD98HC antibodies showed that glucose deprivation caused a decrease in the70∼100 kDa. Glycosylated form of CD98HC, while an approximately 55 kDa low molecular weight form was induced by glucose deprivation. Immunoblot analysis with anti-Mac2BP antibodies showed that the 70 and 90 kDa. Glycosylated forms of Mac2BP decreased and the approximately 55 kDa low molecular weight form was induced by glucose deprivation. The bands corresponding to the lower molecular weight forms of CD98HC and Mac2BP also matched the 55 kDa bands previously identified as induced proteins based on their reaction with CTD110.6 antibodies under glucose deprivation. Immunoblot analysis following the time course of glucose deprivation responses showed that the levels of the lower molecular weight forms of the four proteins were clearly increased at 6 h after the treatment, i.e. at the same time that an increase was previously noted for proteins identified by their reaction with CTD110.6 antibodies (Figure 1b). The immunoprecipitation analysis showed that CTD110.6 antibodies reacted with all four of the lower molecular weight forms that were immunoprecipitated by their specific antibodies, respectively, but not with the mature glycosylated forms. Knockdown of *ORP150* with siRNA specifically decreased the expression of the 120 kDa ORP150 protein, which was previously identified using the CTD110.6 antibodies under glucose deprivation (Figure S4). These results demonstrated that the lower molecular weight forms of ORP150, Laminin β3, CD98HC, and Mac2BP were induced by glucose deprivation, and that CTD110.6 antibodies also reacted with them under glucose deprivation.

**Figure 3:**
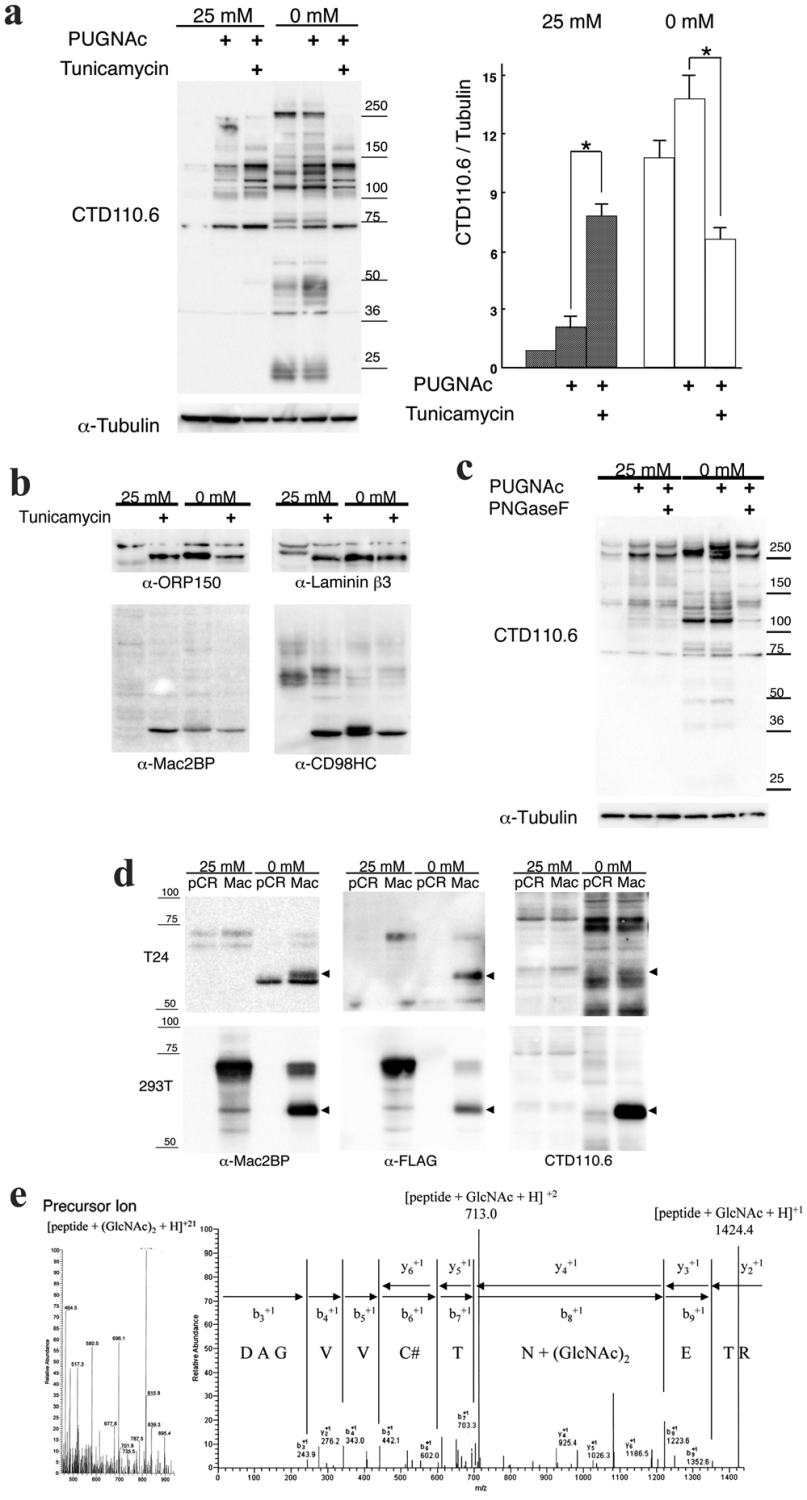
The proteins induced by glucose deprivation were modified with *N*-GlcNAc_2_. **a**, The effects of tunicamycin treatment on the expression of proteins that reacted with CTD110.6 antibodies under glucose deprivation. The left panel shows immunoblots for CTD110.6 and anti-α-tubulin antibodies. The right panel shows a quantitative analysis of reactivity with the CTD110.6 antibody and with an anti-OGT antibody, normalized to the anti-α-tubulin signal for untreated samples in high-glucose medium. Error bars represent standard error from three experiments. * represents p<0.01. **b**, An immunoblot showing effects of tunicamycin treatment on the four induced proteins. **c**, An immunoblot showing the effects of the treatment of PNGase F treatment on the supernatants from T24 cells that were cultured in glucose deprivation medium. **d**, An immunoblot showing the effects of Mac2BP over-expression in T24 cells and 293T cells. **e**, The LC/MS/MS spectrum analysis for the peptides derived from over-expressed Mac2BP protein.

We next examined the effects of tunicamycin, which inhibits *N*-glycosylation, on expression of the induced proteins (Figure 3a). Tunicamycin treatment increased the expression of basal proteins in high-glucose medium with PUGNAc, as previously described [13]. These results were reasonable, as the pool of UDP-GlcNAc was increased by the inhibition of tunicamycin, so more was available for *O*-GlcNAcylation. When the cells were incubated in glucose deprivation medium, tunicamycin treatment increased expression of the basal proteins, similar to the response in the high glucose conditions with PUGNAc. However, expression of the induced proteins disappeared following tunicamycin treatment, unlike the basal proteins. ORP150, Laminin β3, CD98HC, and Mac2BP were detected as their naked forms following tunicamycin treatment in both high glucose medium and in glucose deprivation medium (Figure 3b). The molecular weights of these proteins, as detected by CTD110.6 antibodies following glucose deprivation, were slightly higher than the bands produced following tunicamycin treatment. These tunicamycin effects were observed also in HepG2 (Figure S5) cells and Neuro-2a cells (Figure S6). When the supernatants derived from T24 cells incubated in glucose deprivation medium were treated with Peptide-N-glycosidase F (PNGaseF), which removes all *N*-glycans from peptides, the bands corresponding to the induced proteins disappeared, although the bands corresponding to the basal proteins did not change (Figure 3c). These results suggested that the induced proteins contained *N*-linked sugars.

The induced proteins were analyzed by LC/MS/MS in order to further characterize the sugar residues. FLAG-tagged Mac2BP proteins were over-expressed in T24 and 293T cells (Figure 3d). In both cases, these proteins reacted with CTD110.6 antibodies under glucose deprivation, suggesting that they had been modified with sugar residues. FLAG-tagged Mac2BP proteins that were over-expressed in 293T cells were concentrated by α-FLAG beads and analyzed by LC/MS/MS, and peptides containing residues modified with GlcNAc_2_ were found (Figure 3e). The Mac2BP protein sequence contains seven potential *N*-linked glycosylation sites (N-X-S/T), and *N*-GlcNAc_2_ modification was found at five of these sites (Table S2). This result suggested that CTD110.6 antibodies reacted with *N*-GlcNAc_2_-modifed proteins induced by glucose deprivation.

**Figure 4:**
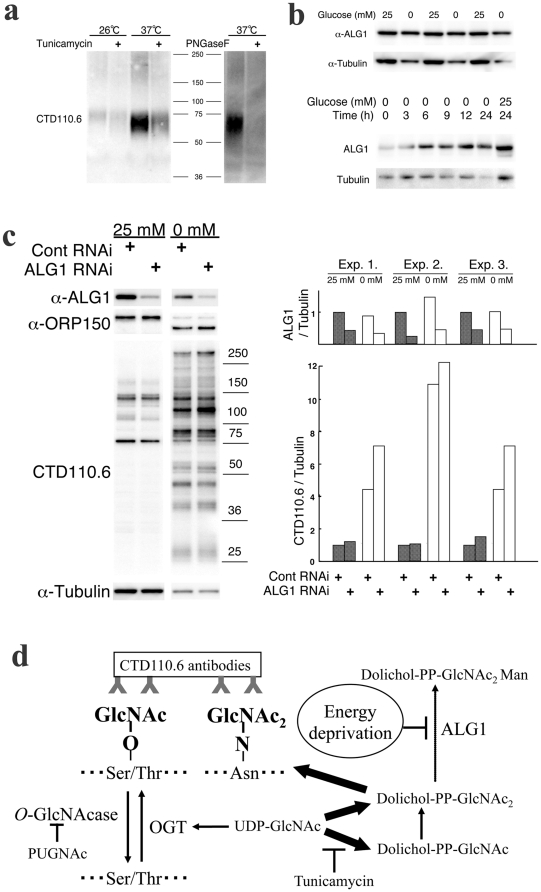
*N*-GlcNAc_2_ produced by repression of Alg1 cross-reacts with CTD110.6 antibodies. **a**, An immunoblot showing CTD110.6 antibody of *Alg1*-ts-mutant yeast culture medium. **b**, An immunoblot showing anti-Alg1 antibody reaction over the time course during incubation of T24 cells in glucose deprivation medium (0 mM glucose). An anti-α-tubulin antibody was used as an internal control. **c**, The effects of treatment with siRNA specific for *Alg1* on the expression of proteins that reacted with CTD110.6 antibodies under glucose deprivation. The left panel shows an immunoblot for CTD110.6, anti-ALG1, anti-ORP150 and anti-α-tubulin antibodies. The right panel shows a quantitative analysis of reactivity with the CTD110.6 antibody and with ALG1 in three experiments, all normalized to the anti-α-tubulin signal for untreated samples in high-glucose medium. **d**, A schematic summary of all of the experimental results and the hypothesis.

The existence of *N*-GlcNAc_2_-modifed proteins has been reported in the yeast ts-mutant K57-6C [14]. This mutant could not add a mannose residue to dolichol-PP-GlcNAc_2_, as indicated by a loss of ALG1 activity and secretion of *N*-GlcNAc_2_-modifed exoglucanase following a temperature shift from 25 to 37°C. *N*-GlcNAc_2_-modifed exoglucanase protein was detected in the yeast culture medium at 37°C by CTD110.6 antibodies (Figure 4a). This signal decreased following tumicamycin treatment of the 37°C culture and it disappeared following PNGaseF treatment of the medium supernatant. These results suggested that CTD110.6 antibodies reacted with the *N*-GlcNAc_2_-modifed proteins that were induced by the loss of ALG1 activity.

In yeast, the expression of ALG1 is decreased when the carbon source is depleted [15]. However, in T24 cells, the ALG1 band shifted to a slightly higher molecular weight and this shifted band appeared 3 h after glucose deprivation, before the production of *N*-GlcNAc_2_-modifed proteins was observed (Figure 4b). This suggested that the ALG1 enzyme activity may have been lost. We examined the effects of siRNA specific for *ALG1*on the production of *N*-GlcNAc_2_-modifed proteins (Figure 4c). Under high-glucose medium conditions, the expression of proteins reacted with the *O*-GlcNAc specific antibody CTD110.6 and the expression of *N*-GlcNAc_2_-modifed ORP150 hardly increased. However, under glucose deprivation, the expression of proteins reacted with the *O*-GlcNAc specific antibody CTD110.6 and the expression of *N*-GlcNAc_2_-modifed ORP150 some increased. This result suggested that inhibition of ALG1 induced the production of *N*-GlcNAc_2_-modifed proteins under glucose deprivation. The slight effects of siRNA may have been due to a loss of ALG1 enzyme activity caused by modification of ALG1 under glucose deprivation. The partial knock down by siRNA of *ALG1* in the high-glucose medium may have caused incomplete inhibition of *N*-GlcNAc_2_-modifed protein production.

## Discussion

The experiments described here clearly indicate that *N*-GlcNAc_2_-modifed proteins induced under glucose deprivation cross-reacted with the antibody CTD110.6, indicating that this antibody is not actually specific for *O*-GlcNAc-modified proteins. A schematic summary of all of the experimental results and the hypothesis is presented in Figure 4d. Previous reports have demonstrated the specificity of CTD110.6 antibodies for *O*-linked derivatives, but their possible specificity for *N*-linked derivatives containing *N*-GlcNAc_2_ was not evaluated [16]. *N*-GlcNAc may mimic S/T and thus ‘deceive’ CTD110.6 antibodies as it attaches the second GlcNAc with an *O*-type linkage. Previous paradoxical reports that glucose deprivation increased protein *O*-GlcNAcylation in some cancer cells may now be explained by the present study. Our results (Figure 3a, 3b, and Figure S5, S6) suggest that *N*-GlcNAc_2_-modifed proteins, with which CTD110.6 antibodies reacted under glucose deprivation, are produced by T24 cells as well as by some cancerous cells. Therefore, we proposed that the proteins previously classified as *O*-GlcNAc-modified proteins according to their reactivity with CTD110.6 antibodies must be re-examined to determine whether they are actually *O*-GlcNAc- or *N*-GlcNAc_2_-modifed proteins. The *O*-GlcNAc specific antibody, RL-2, is not *N*-GlcNAc_2_-modifed proteins (Figure S2), so RL-2 antibodies may be more specific for *O*-GlcNAc-modifed proteins than CTD110.6 antibodies. Our work has demonstrated that production of *N*-GlcNAc_2_-modifed proteins will induced by inhibiting the addition of a mannose residue to dolichol-PP-GlcNAc_2_ using the yeast *Alg1* ts-mutant. In nature, *Plasmodium falicparum* lacks the *Alg1*gene and produces *N*-GlcNAc_2_-modifed proteins [17]. Congenital Disorders of Glycosylation (CDG) is N-glycosylation disorder induced by mutations of genes in dolichol-linked oligosaccharide biosynthetic pathway [18]. Type Ik patients had mutations of the *Alg1* gene and accumulated dolichol-PP-GlcNAc_2_ [19]. This suggested that these patients produced *N*-GlcNAc_2_-modifed proteins, similar to the phenomenon observed for the yeast *Alg1* ts-mutant. Type Ia, the largest group of CDG patients, had mutations of the Phosphomannomutase 2 (*PMM2*) gene, which encodes a key enzyme of GDP-mannose synthesis [18]. GDP-mannose is a substrate for ALG1, so inhibition of PMM2 enzyme activity will accumulate dolichol-PP-GlcNAc_2_ and produce *N*-GlcNAc_2_-modifed proteins. Glucose deprivation will inhibit dolichol-linked oligosaccharide biosynthesis like the defection of CDG patients. Our digital expression analysis for the time course of glucose deprivation by next-generation sequencing showed that the expression level of Serum and Glucocorticoid-regulated kinase (Sgk1), which inhibits PMM2 enzyme activity [20], was up-regulated tenfold by glucose deprivation (data in preparation). This suggests that the synthesis of GDP-mannose was inhibited by glucose deprivation. It is likely that inactivation of both ALG1 and GDP-mannose synthesis was induced under glucose deprivation and that this contributed to the production of *N*-GlcNAc_2_-modifed proteins. Therefore, *N*-GlcNAc_2_-modifed proteins may not be produced in high-glucose medium only through inhibition by *ALG1* siRNA. The addition of glucosamine under low glucose conditions induced the typical *O*-GlcNAc-modified proteins but not *N*-GlcNAc_2_-modifed proteins [9]. An adequate supply of sugars such as glucose and glucosamine will overcome inhibition of GDP-mannose synthesis and will not induce the production of *N*-GlcNAc_2_-modified proteins. The production of *N*-GlcNAc_2_-modified proteins in T24 and some cancer cell line cells may require the deprivation of sugar. We suggest that the repression of mature *N*-linked glycoproteins due to increased levels of *N*-GlcNAc_2_-modifed proteins is a newly recognized pathway for effective use of sugar under stress and deprivation conditions. Further research is needed to clarify the physiological and pathological roles of *N*-GlcNAc_2_-modifed proteins, and the results of these studies will be applicable across a wide range of fields.

## Materials and Methods

### Cell lines and cell culture conditions

The human bladder cancer cell line, T24 [21] and human embryonic kidney cell line, 293T [22] were usually cultured in the high-glucose version of Dulbecco's modified Eagle's medium (DMEM, Nacalai Tesque, Kyoto, Japan), which contained 25 mM glucose and 1 mM sodium pyruvate, supplemented with 10% fetal calf serum (FCS), penicillin (100 U/ml), and streptomycin (100 µg/ml) at 37°C in a humidified 5% CO_2_ atmosphere. In the experimental culture, cells were seeded in high-glucose medium and then treated with or without transfection of plasmid DNA or small interference RNA (siRNA) at day 1. The culture medium was then replaced on day 2 with fresh high-glucose medium (25 mM glucose) or with energy deprivation medium, which was depleted of glucose and sodium pyruvate (0 mM glucose, Invitrogen, Carlsbad, CA), and supernatants were collected on day 3. The treatments with PUGNAc (100 µM) and tunicamycin (2 µg/ml) were carried out when the medium was replaced on day 2. *Saccharomyces cerevisiae* strain K57-6C (MATa, alg1-1, ura3-52) was purchased from ATTC. Yeast cells were maintained in YEPD medium. For the production of external exoglucanase, cells were grown in liquid minimal medium supplemented with uracil plus adenine at 26 and 37°C as described [14].

### Plasmids and transfection

We amplified a human gene for *Mac2BP* from T24 cells by PCR, using a pair of human *Mac2BP*-specific primers: MACS1 (5′-AGGCACGGCCATGACCCCTC-3′, nucleotide 170–189) and MACA2 (5′-GTCCACACCTGAGGAGTTGGTC-3′, nucleotide 1914–1893), which corresponded to the nucleotide sequence of the human *Mac2BP* gene (ENST00000262776). The amplified PCR fragments were cloned into the *EcoR*I/blunt site of the pCR3.1-FLAG-CT expression vector [23]. The pCR3.1-FLAG-CT was constructed by a synthesized FLAG tag cassette introduced into the *EcoR*I-*Pst*I/blunt sites of the pCR3.1 expression vector, and a stop codon at the C-terminus of the FLAG tag sequence was introduced by this joining. DNA sequencing of the PCR products was performed by the dideoxy chain termination method using an ABI PRISM 3130xl Genetic Analyzer (Applied Biosystems, Foster City, CA, USA). Plasmids were prepared using PureLink HiPure Plasmid DNA Purification Kits (Invitrogen). Transfection of plasmids was carried out in 90-mm dishes using Lipofectamine 2000 reagent (Invitrogen) according to the manufacturer's protocol.

### siRNA

siRNA targeting human *OGT* (L-019111-00-0005) and scrambled control (D-001810-01-05) RNA duplexes were purchased from Dharmacon, Inc. (Lafayette, CO). siRNAs targeting human *Alg1* (NM_019109_stealth_372) RNA duplexes were purchased from Invitrogen. Cells were transfected with RNA duplexes using Lipofectamine RNAiMAX reagents (Invitrogen) following the manufacturer's protocol.

### Antibodies

Anti-*O*-GlcNAc (#MMS-248R, CTD110.6) IgM monoclonal antibody was purchased from Covance (Berkeley, CA). Anti-CD98HC (#9160, H-300), anti-OGT (#32921, H-300), and anti-Laminin β-3 (#20775, H-300) antibodies were purchased from Santa Cruz Biotechnology (Santa Cruz, CA). Anti-Mac2BP (#710281-1-AP) and anti-ALG1 (#12872-1-AP) antibodies were purchased from Protein Tech Group (Chicago, Ill.). Anti-ORP150 antibody (#10301, monoclonal) was purchased from Immuno-Biological Laboratories (Takasaki, Japan). Anti-α-tubulin (#T9026, DM1A) and anti-FLAG (#F3165, M2) monoclonal antibodies were purchased from Sigma-Aldrich (St. Louis, MO).

### Immunoblotting

Cells were lysed in Laemmli-SDS buffer, subjected to SDS-polyacrylamide gel electrophoresis, and electro-transferred to membrane filters (Immuno-Blot PVDF membranes, Bio-Rad Laboratories, Richmond, CA). The filters were incubated with a primary antibody in TBS-T (10 mM Tris-HCl, pH 7.6, 150 mM sodium chloride, 0.1% Tween20) containing 2% bovine serum albumin (BSA) overnight and incubated for 1 hour in horseradish peroxidase-conjugated anti-mouse, anti-rabbit (GE Healthcare, Buckinghamshire, UK), or anti-mouse IgM (Sigma-Aldrich) diluted 1∶5,000 in TBS-T containing 2% BSA. Immunoreactivity was detected using the ECL system (GE Healthcare) with LAS4000 (Fujifilm, Tokyo, Japan) and quantified with Multi gauge (Fujifilm), using an anti-α-tubulin antibody as an internal control. All quantification analyses were performed in triplicate.

### Immunoprecipitation

Cells were lysed in Brij lysis buffer containing 20 mM Tris-HCl (pH 7.4), 150 mM NaCl, 1% Brij 98 (Sigma-Aldrich), a protease inhibitor cocktail (Nakalai Tesque), *O*-GlcNAcase inhibiter, O-(2-acetamide-2-deoxy-D-glucopyranosilidene)amino N-phenylcarbamate (PUGNAc; Toronto Research Chemicals, Ontario, Canada), and a phosphatase inhibitor cocktail (Sigma-Aldrich) and were centrifuged for 30 minutes at 10,000×g at 4°C. The supernatant was incubated with the specific antibody at 4°C for 1 hour. The immunocomplexes were bound to protein-G sepharose for 1 hour at 4°C and washed five times with TBS-T. The proteins bound to the resin were eluted by adding 2× Laemmli-SDS sample buffer, to a final concentration of 62.5 mM Tris-HCl (pH 6.8), 10% glycerol, 5% 2-mercaptoethanol, 2% sodium dodecyl sulfate (SDS), and 0.01% bromophenol blue, and the samples were boiled for 5 minutes. After centrifugation at 10,000×g for 2 minutes, the supernatants were analyzed by immunoblotting.

### Identification of the proteins by LC/MS/MS analyses

T24 cells were lysed in Brij lysis buffer and centrifuged for 30 minutes at 10,000×g at 4°C. The supernatants were incubated with the anti-*O*-GlcNAc CTD110.6 IgM monoclonal antibody at 4°C for 1 hour and for an additional 1 hour with added anti-mouse IgM antibody. The immunocomplexes were bound to protein-G sepharose for 1 hour at 4°C and washed five times with TBS-T. The proteins bound to the agarose were eluted by adding 2× Laemmli-SDS sample buffer and subjected to SDS-polyacrylamide gel electrophoresis. To identify the CTD110.6 antibody-reacted bands, the electro-transferred membrane filter was both stained with DeepPurple (GE Helthcare) and immunoblotted with CTD110.6 antibody. Each identified band, which was stained with SproRuby in another gel, was digested in the gel by modified trypsin (Promega, Madison, WI) as described previously [24]. The extracted samples were analyzed using the LC/MS/MS system with Paradigm MS4 (AMR, Tokyo, Japan), Finnigan LCQ Advantage (Thermo Fisher Scientific, Waltman, MA), and Bioworks analyzer soft (Thermo Fisher Scientific). FLAG-tagged Mac2BP proteins in 293T cells were concentrated with anti-FLAG affinity agarose (Sigma-Aldrich) for LC/MS/MS analysis.

### PNGase F treatments

T24 cells were lysed in the solubilization buffer containing 1% SDS, 20 mM sodium phosphate (pH 6.0), 10 mM EDTA, 1% 2-mercaptoethanol, heated for 2 minutes, diluted using a dilution buffer containing 0.5% NP40, 50 mM sodium phosphate (pH 6.0), 10 mM EDTA, 1% 2-mercaptoethanol, and then treated with 1/15 U PNGase F for 5 hours. The supernatants of the yeast cell cultures were concentrated by Microcon YM-10 (Millipore, Billerica, MA) and treated as described above.

### Statistics

Results of experiments are represented as mean ± S.E. Each mean represents data from at least three independent experiments. The Student's *t* test (two-tail) was used to compare differences between groups.

## Supporting Information

Figure S1 **The effects of the treatment with 10mMGlcNAc on the expression of proteins that reacted with CTD110.6 antibodies under glucose deprivation.** The left panel shows an immunoblot for CTD110.6 and anti-α-tubulin antibodies. The right panel shows an immunoblot for CTD110.6 with 10mMGlcNAc and anti-α-tubulin antibodies.
(TIF)

Figure S2 **The reactivity of the induced proteins, that reacted with CTD110.6 antibodies under glucose deprivation, with** ***O*** **-GlcNAc-specific antibody RL2.** The left panel shows an immunoblot for RL2 (Santa Cruz Biotechnology) and anti-α-tubulin antibodies. The right panel shows a quantitative analysis of reactivity with the RL2 antibody, normalized to the anti-α-tubulin signal for untreated samples in high-glucose medium. Error bars represent standard error from three experiments.
(TIF)

Figure S3 **The reactivity of the induced proteins, that reacted with CTD110.6 antibodies under glucose deprivation, with Click-iT** ***O*** **-GlcNAc Enzymatic labeling system.** Cell extract collected from each one day culture of T24 cell by solubilizing with Brij lysis buffer. *O*-GlcNAc-modified proteins were labeled with Click-iT *O*-GlcNAc Enzymatic labeling system (Invitrogen) and Click-iT biotin Glycoprotein Detection Kit (Invitrogen) according to the manufacture's protocol. The left panel shows an immunoblot for CTD110.6 antibodies for cell extract. The right panel shows an immunoblot for anti-biotin antibodies (Cell Signaling Technology) for biotin-labeled samples.
(TIF)

Figure S4 **The effects of the treatment with siRNA specific for** ***ORP150*** **on the expression of proteins that reacted with CTD110.6 antibodies under glucose deprivation.** siRNAs targeting human *ORP150* (NM_001130991_stealth_455) RNA duplexes were purchased from Invitrogen. The left panel shows an immunoblot for CTD110.6, anti-ORP150, and anti-a-tubulin antibodies. The right panel shows a quantitative analysis of reactivity with the ORP150 antibody, normalized to the anti-α-tubulin signal for untreated samples in high-glucose medium. The anti-ORP150 antibody reacted with ORP150 and with GPR170 (mature form with glycosylation) proteins. Error bars represent standard error from three experiments.
(TIF)

Figure S5 **The effects of tunicamycin treatment on the expression of proteins that reacted with CTD110.6 antibodies under glucose deprivation in HepG2 cells.** The immunoblots are shown for CTD110.6, anti-ORP150, anti-Mac2BP, anti-CD98H, and anti-α-tubulin antibodies.
(TIF)

Figure S6 **The effects of tunicamycin treatment on the expression of proteins that reacted with CTD110.6 antibodies under glucose deprivation in Neuro-2 cells.** The immunoblots are shown for CTD110.6, anti-ORP150, and anti-α-tubulin antibodies.
(TIF)

Table S1 **Proteins that were induced by glucose deprivation of T24 cells and identified by LC/MS/MS analysis.** The probability score, P, is from a new scoring algorithm in BioWorks that is based on the probability that the peptide is a random match to the spectral data. Values of p<0.001 were considered statistically significant. The final score, Sf, indicates how good the protein and peptide match is between the experimental MS/MS data and the theoretical data. The Sf score combines various scores into one final score. Values of Sf >0.40 were considered statistically significant.
(TIF)

Table S2 **The** ***N*** **-GlcNAc_2_-peptides from Mac2BP proteins that were induced in T24 cells under glucose deprivation and identified by LC/MS/MS analysis.** The probability score, P, is from a new scoring algorithm in BioWorks that is based on the probability that the peptide is a random match to the spectral data. The final score, Sf, indicates how good the protein and peptide match is between the experimental MS/MS data and the theoretical data. The Sf score combines various scores into one final score. Each peptide score was derived using the maximum amount of data available.
(TIF)
