# Supplementary figures and images for: O-GlcNAc-Specific Antibody CTD110.6 Cross-Reacts with N-GlcNAc2-Modified Proteins Induced under Glucose Deprivation
Source: PLoS One. 2011 Apr 19;6(4):e18959. doi: 10.1371/journal.pone.0018959 (PMC3079744; doi:10.1371/journal.pone.0018959)

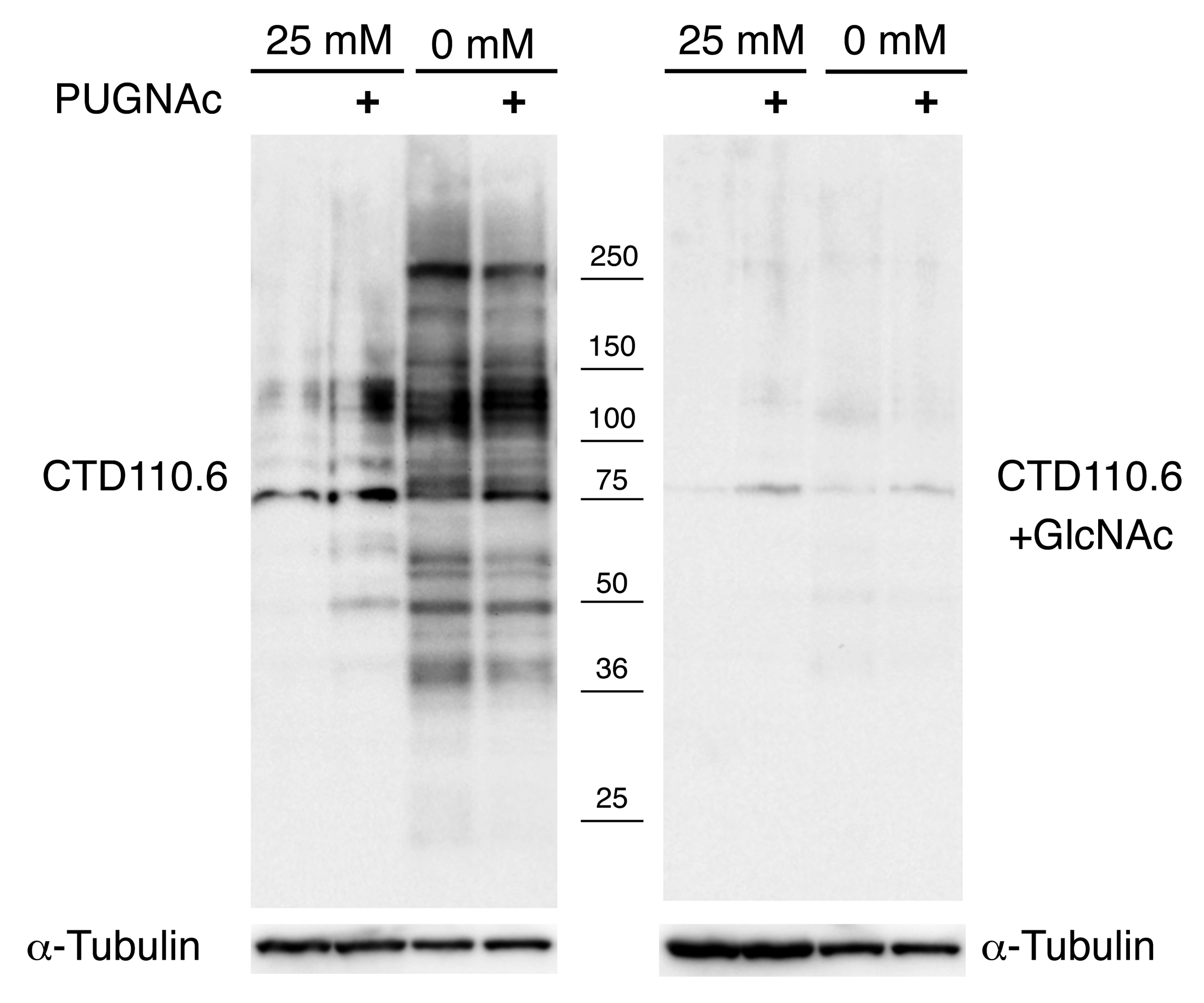

Supplement: Figure S1 — The effects of the treatment with 10mMGlcNAc on the expression of proteins that reacted with CTD110.6 antibodies under glucose deprivation. The left panel shows an immunoblot for CTD110.6 and anti-α-tubulin antibodies. The right panel shows an immunoblot for CTD110.6 with 10mMGlcNAc and anti-α-tubulin antibodies. (TIF) [file pone.0018959.s001.tif]

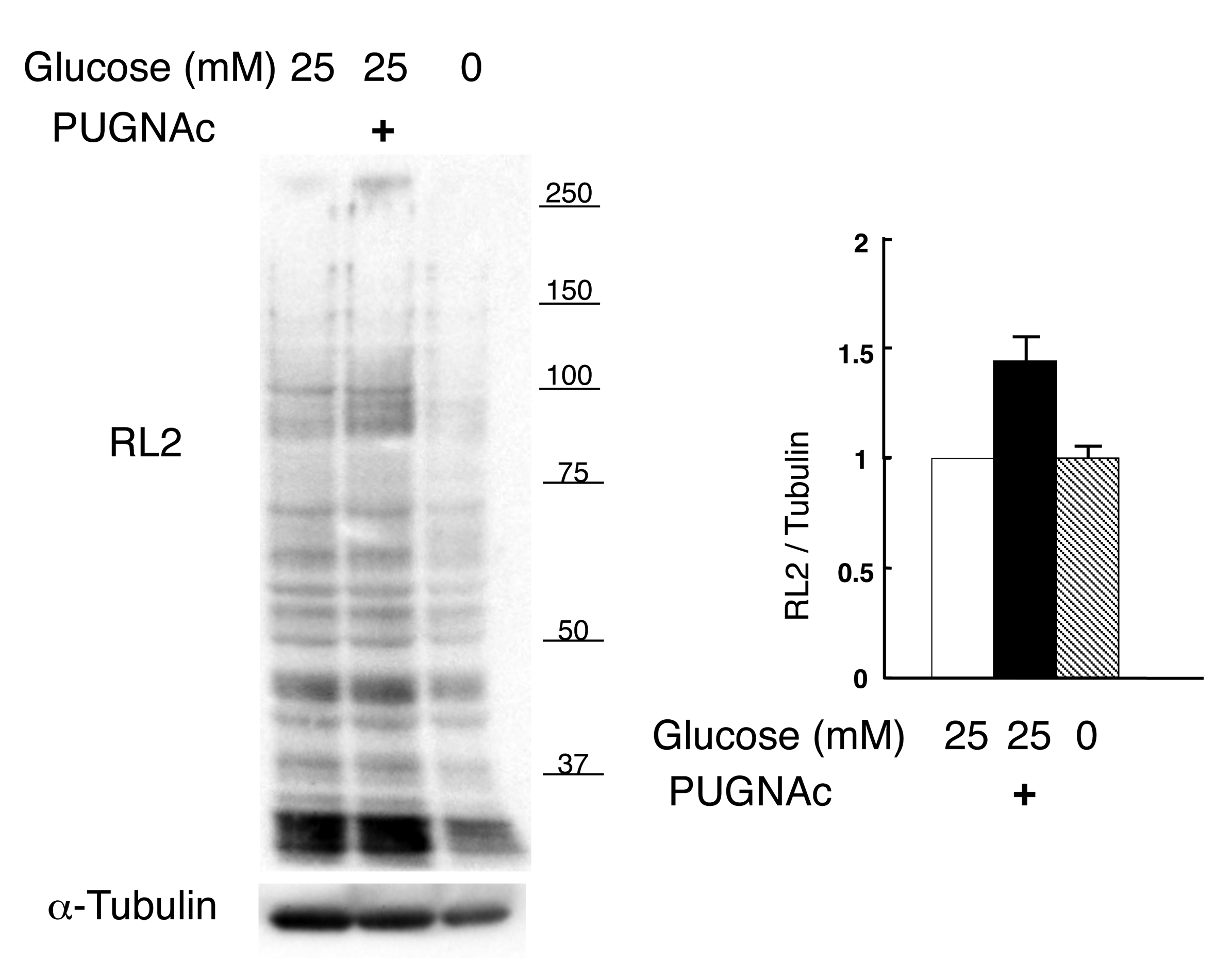

Supplement: Figure S2 — The reactivity of the induced proteins, that reacted with CTD110.6 antibodies under glucose deprivation, with O -GlcNAc-specific antibody RL2. The left panel shows an immunoblot for RL2 (Santa Cruz Biotechnology) and anti-α-tubulin antibodies. The right panel shows a quantitative analysis of reactivity with the RL2 antibody, normalized to the anti-α-tubulin signal for untreated samples in high-glucose medium. Error bars represent standard error from three experiments. (TIF) [file pone.0018959.s002.tif]

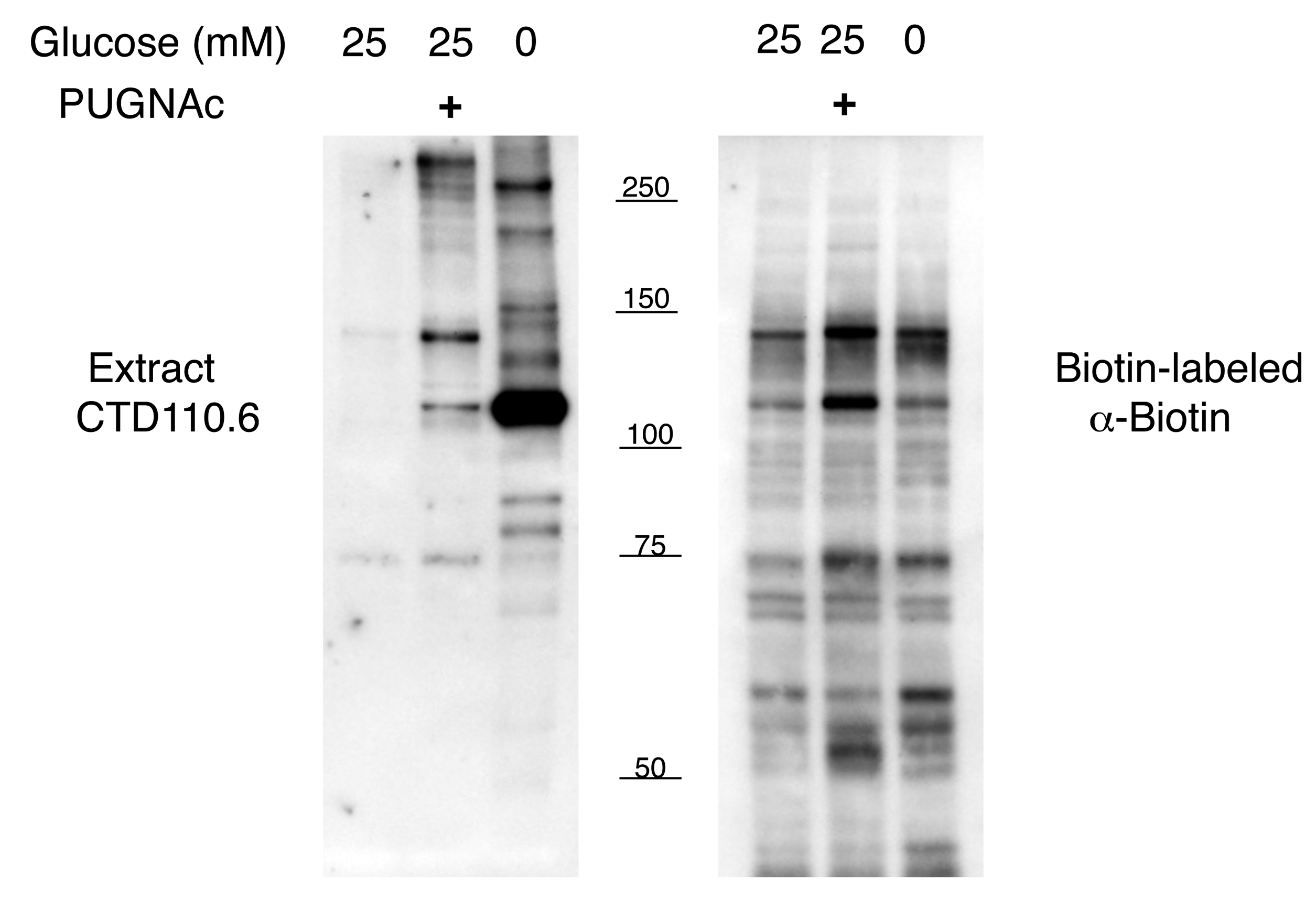

Supplement: Figure S3 — The reactivity of the induced proteins, that reacted with CTD110.6 antibodies under glucose deprivation, with Click-iT O -GlcNAc Enzymatic labeling system. Cell extract collected from each one day culture of T24 cell by solubilizing with Brij lysis buffer. O-GlcNAc-modified proteins were labeled with Click-iT O-GlcNAc Enzymatic labeling system (Invitrogen) and Click-iT biotin Glycoprotein Detection Kit (Invitrogen) according to the manufacture's protocol. The left panel shows an immunoblot for CTD110.6 antibodies for cell extract. The right panel shows an immunoblot for anti-biotin antibodies (Cell Signaling Technology) for biotin-labeled samples. (TIF) [file pone.0018959.s003.tif]

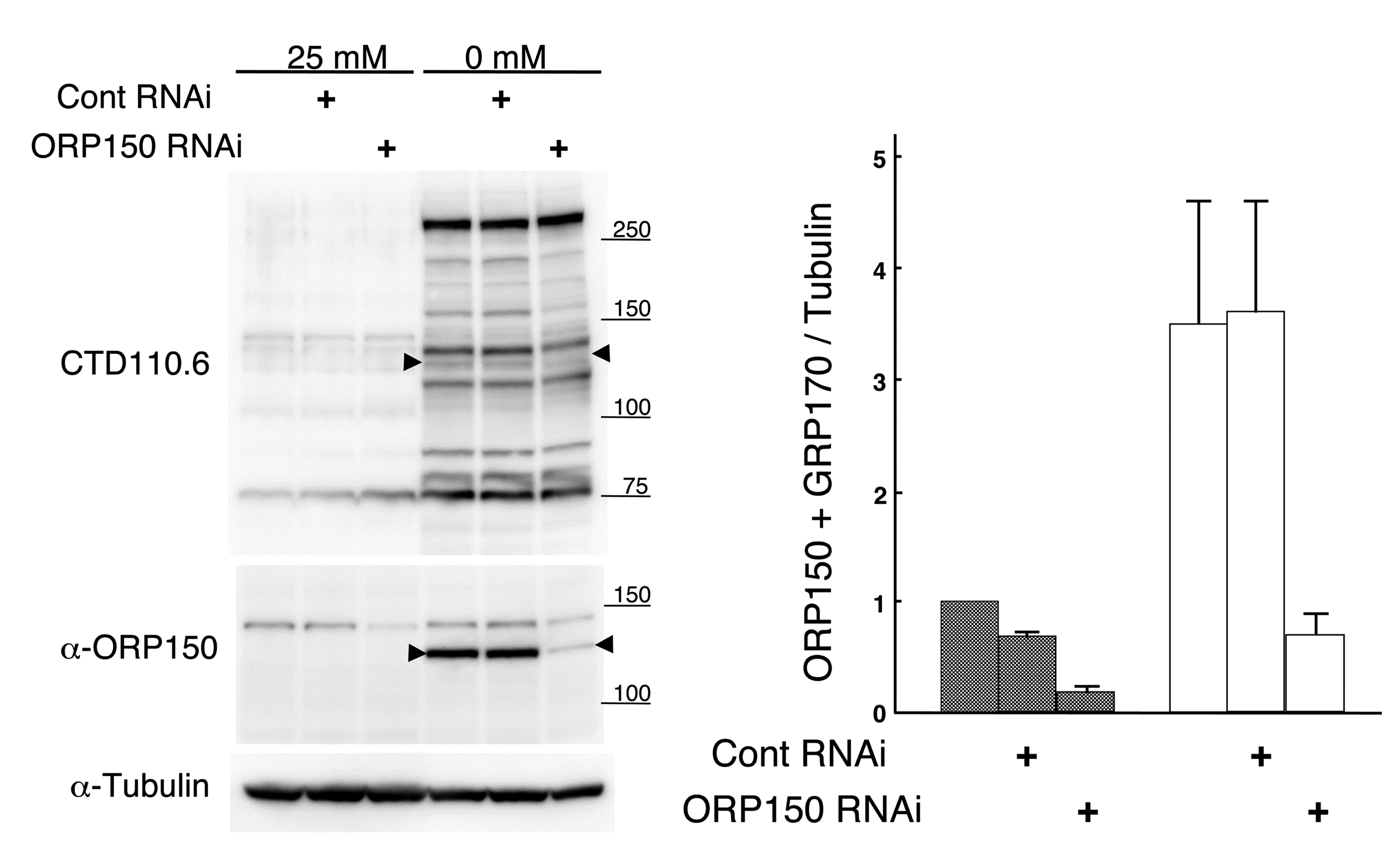

Supplement: Figure S4 — The effects of the treatment with siRNA specific for ORP150 on the expression of proteins that reacted with CTD110.6 antibodies under glucose deprivation. siRNAs targeting human ORP150 (NM_001130991_stealth_455) RNA duplexes were purchased from Invitrogen. The left panel shows an immunoblot for CTD110.6, anti-ORP150, and anti-a-tubulin antibodies. The right panel shows a quantitative analysis of reactivity with the ORP150 antibody, normalized to the anti-α-tubulin signal for untreated samples in high-glucose medium. The anti-ORP150 antibody reacted with ORP150 and with GPR170 (mature form with glycosylation) proteins. Error bars represent standard error from three experiments. (TIF) [file pone.0018959.s004.tif]

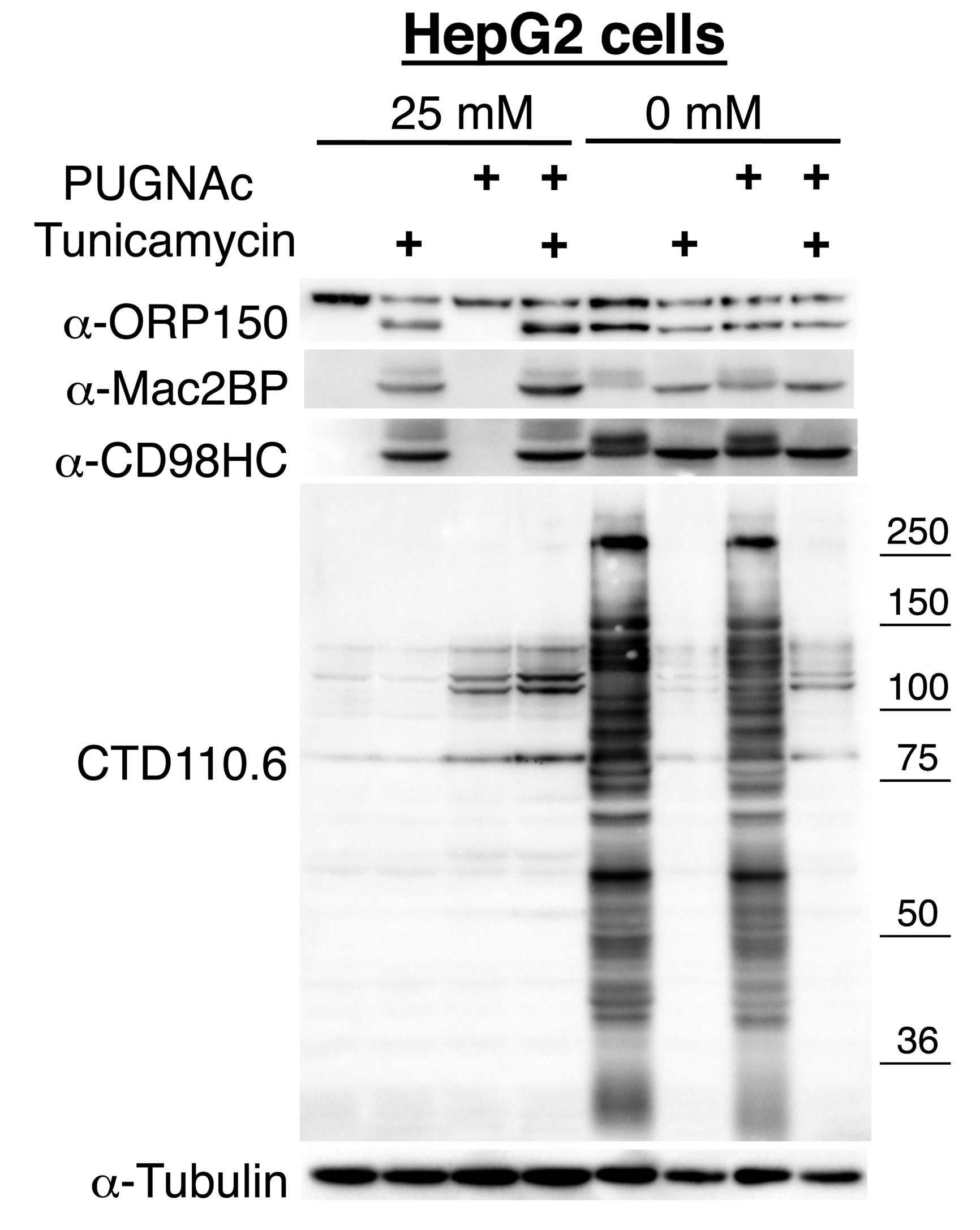

Supplement: Figure S5 — The effects of tunicamycin treatment on the expression of proteins that reacted with CTD110.6 antibodies under glucose deprivation in HepG2 cells. The immunoblots are shown for CTD110.6, anti-ORP150, anti-Mac2BP, anti-CD98H, and anti-α-tubulin antibodies. (TIF) [file pone.0018959.s005.tif]

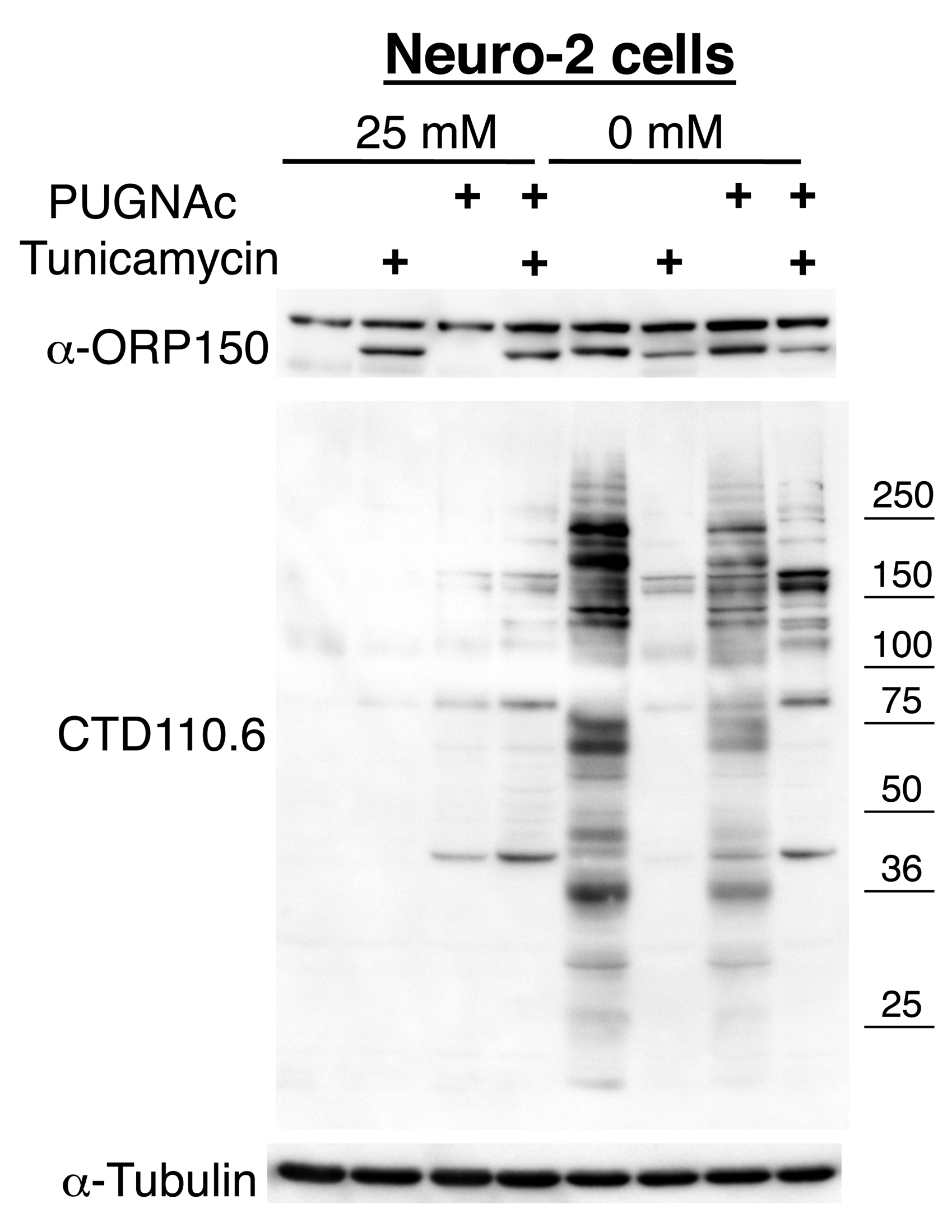

Supplement: Figure S6 — The effects of tunicamycin treatment on the expression of proteins that reacted with CTD110.6 antibodies under glucose deprivation in Neuro-2 cells. The immunoblots are shown for CTD110.6, anti-ORP150, and anti-α-tubulin antibodies. (TIF) [file pone.0018959.s006.tif]

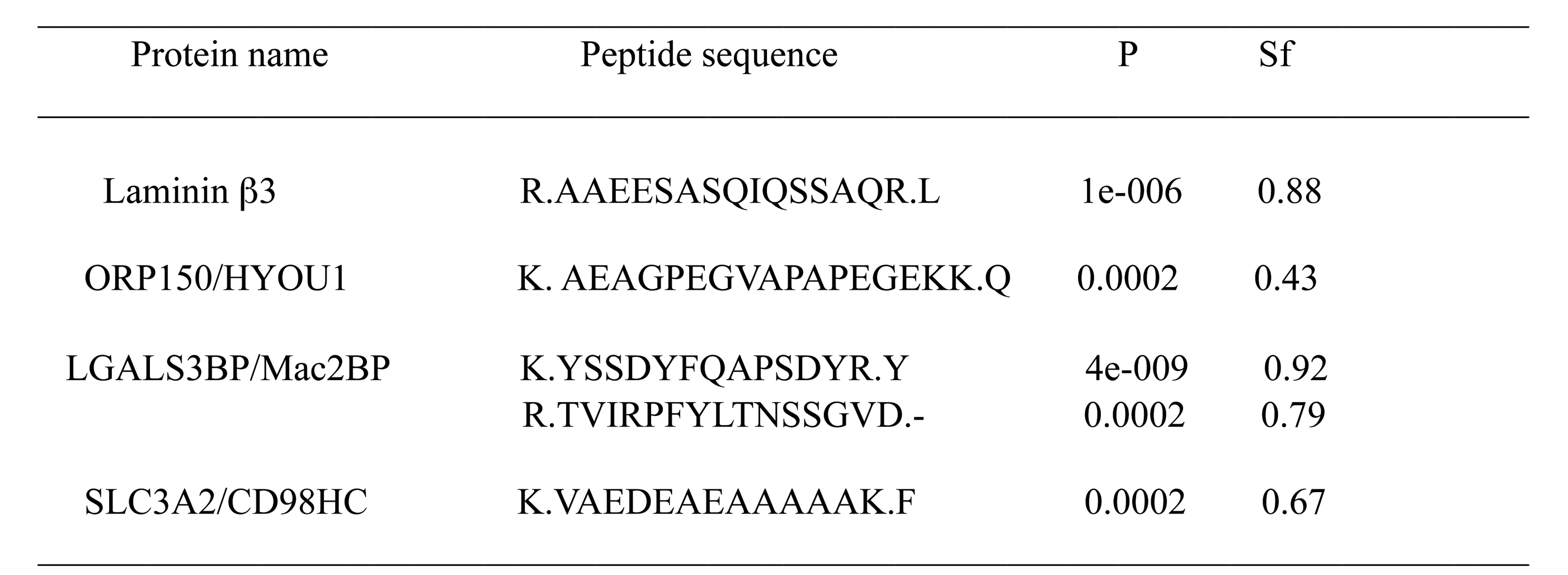

Supplement: Table S1 — Proteins that were induced by glucose deprivation of T24 cells and identified by LC/MS/MS analysis. The probability score, P, is from a new scoring algorithm in BioWorks that is based on the probability that the peptide is a random match to the spectral data. Values of p<0.001 were considered statistically significant. The final score, Sf, indicates how good the protein and peptide match is between the experimental MS/MS data and the theoretical data. The Sf score combines various scores into one final score. Values of Sf >0.40 were considered statistically significant. (TIF) [file pone.0018959.s007.tif]

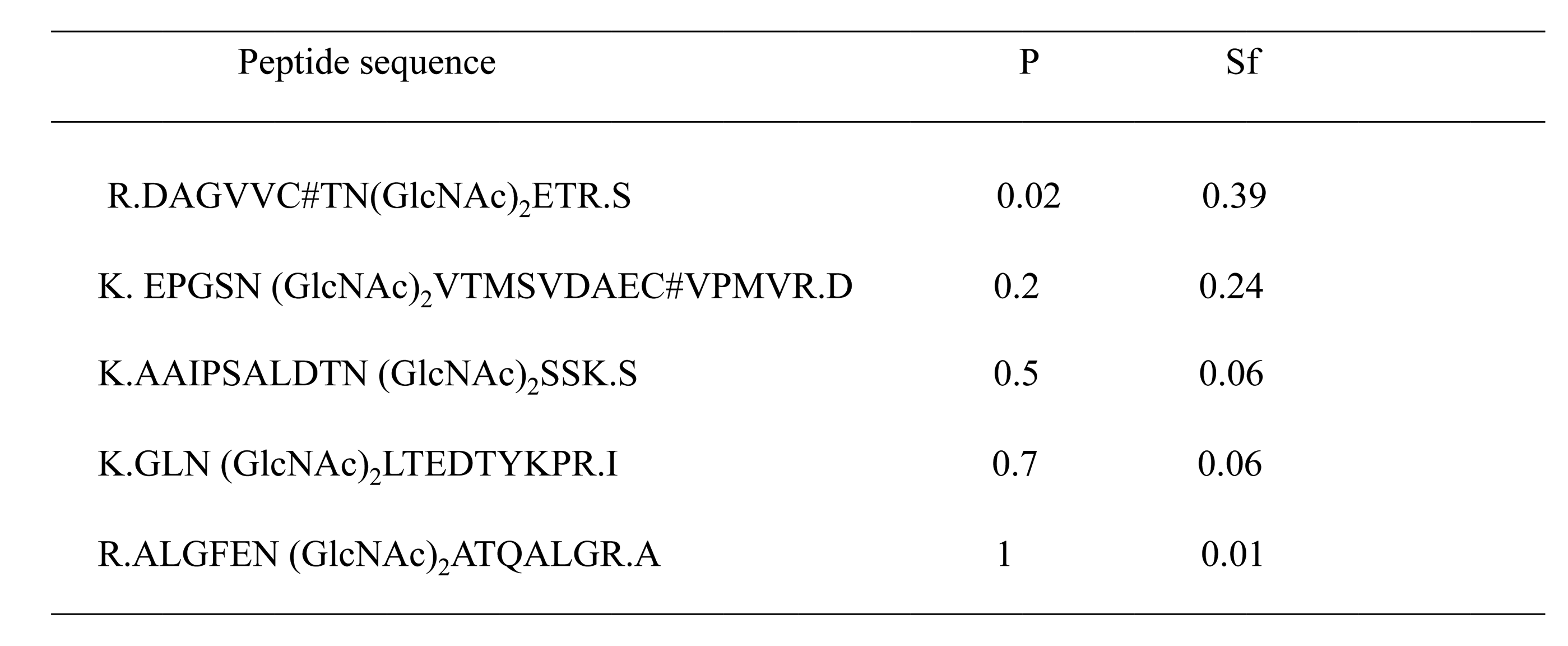

Supplement: Table S2 — The N -GlcNAc2-peptides from Mac2BP proteins that were induced in T24 cells under glucose deprivation and identified by LC/MS/MS analysis. The probability score, P, is from a new scoring algorithm in BioWorks that is based on the probability that the peptide is a random match to the spectral data. The final score, Sf, indicates how good the protein and peptide match is between the experimental MS/MS data and the theoretical data. The Sf score combines various scores into one final score. Each peptide score was derived using the maximum amount of data available. (TIF) [file pone.0018959.s008.tif]
